# Problems with theories that equate consciousness with information or information processing

**DOI:** 10.3389/fnsys.2014.00225

**Published:** 2014-11-25

**Authors:** Susan Pockett

**Affiliations:** School of Psychology, University of AucklandAuckland, New Zealand

**Keywords:** consciousness, process theories, information, Chalmers, Tononi, McFadden, information processing

Attempts to augment the function of the human brain inevitably involve in some way what Block (1995) calls phenomenal consciousness—bodily sensations and perceptual experiences—the redness of a strawberry, the smell of newly-baked bread. At present there is no consensus among scientists about what such sensory experiences are. This Opinion piece points out some problems with one of the major theatrical viewpoints on that question.

## Classification of theories of consciousness

The oldest classification system has two major categories, dualist and monist. Dualist theories equate consciousness with abstracta. Monist (aka physicalist) theories equate it with concreta. The Stanford Encyclopedia of Philosophy approaches the task of defining abstracta and concreta by the ancient method of providing examples and letting the reader work it out for themselves: it says “Some clear cases of abstracta are classes, propositions, concepts, the letter ‘A’, and Dante's Inferno. Some clear cases of concreta are stars, protons, electromagnetic fields, the chalk tokens of the letter ‘A’ written on a certain blackboard, and James Joyce's copy of Dante's Inferno.”

A more recent classification (Atkinson et al., 2000) divides theories of consciousness into process theories and vehicle theories: it says “Process theories assume that consciousness depends on certain functional or relational properties of representational vehicles, namely, the computations in which those vehicles engage. On this view, representational contents are conscious when their vehicles have some privileged computational status, independently of any particular intrinsic property of those vehicles. What counts is ‘what representational vehicles do, rather than what they are’… For vehicle theories, on the other hand, consciousness is determined by intrinsic properties of representational vehicles, independently of any computations in which those vehicles engage.”

The relative number of words devoted to process and vehicle theories in this description hints that at present, process theories massively dominate the theoretical landscape. But how sensible are they really?

## Theories that equate consciousness with information or information processing are dualist

Most process theories identify consciousness with the processing of information. As Velmans (1991) puts it: “For radical behaviorists, all talk of mind could be translated, without scientific loss, into talk about behavior. For the new ‘radical cognitivists’ all talk of mind (including consciousness) can be translated, without scientific loss, into talk about information processing.” In the quarter century since 1991, process theories have become so deeply embedded that the term “radical” no longer applies. Pretty well all cognitive scientists, computationalists and psychologists now think of consciousness in terms of information processing. Indeed, among these groups the information processing paradigm is so prevalent that it is usually not seen as necessary to state it explicitly.

Perhaps as a consequence, it is not widely recognized that the concepts “process,” “information,” and “information processing” are all abstracta. Thus, mapping the new process/vehicle dichotomy onto the old dualist/physicalist axis reveals that process theories (in the sense of theories that equate consciousness with information or information processing *per se*, rather than with any particular physical realization or implementation thereof) are dualist. Philosopher David Chalmers is one of the few process theorists to recognize that his theory is an example of what he calls “naturalistic dualism” (Chalmers, 1996). The word “naturalistic” may have been included in this description in an attempt to make the “dualism” part more acceptable to cognitive scientists, most of whom prefer to see themselves as staunchly scientific physicalists.

## Chalmers' process theory

Chalmers (1996) takes information theory (Shannon, 1948) as his starting point, but immediately generalizes Shannon's two-state “bit” of information to the concept of a multi-state “information space,” defined as an abstract space consisting of a number of information states and a structure of “difference relations” between them. Chalmers then discusses ways in which information states can be realized physically, mentioning thermostats, books, telephone lines, and Bateson's catchy slogan about information's being “a difference that makes a difference,” before proposing as a fundamental principle that “information (in the actual world) has two aspects, a physical and a phenomenal aspect” (Chalmers, 1996, p. 286). On this theory then, information actually *is*—has the property of being—conscious.

One immediate problem with this idea is that it involves a radical redefinition of the word information, slipped in by the back door in the sense that Chalmers never acknowledges that everyone else's definitions are specifically at odds with his.

There are several technical definitions of information, which differ slightly depending on the field of enquiry. In information philosophy, Floridi (2005) says “ ‘information’ is often used to refer to non-mental, user-independent, declarative semantic contents, embedded in physical implementations like databases, encyclopedias, web sites, television programmes and so on… the *Cambridge Dictionary of Philosophy*, for example, defines information thus: ‘an objective (mind independent) entity… Information can be encoded and transmitted, but the information would exist independently of its encoding or transmission’.” Floridi then lists a number of sources that define information as data + meaning, before arguing that truth is also a necessary ingredient (because if information is not truthful, it should more properly be called misinformation or pseudo-information). Other technical definitions exclude even meaning. Classical or Shannon information theory was born out of a need to address the technical problems experienced by Shannon's employer Bell Labs in extracting signals from noise in telephone and telegraph lines, so Shannon (1948) equates information simply with the observation that a particular one out of a defined set of possible messages has been sent from one entity to another—the meaning of the message is explicitly stated to be irrelevant. Cybernetics (Sayre, 1976) later generalizes Shannon's definition to equate information with increased probability, or reduction in uncertainty.

The point is that all of these definitions take information itself as an objective, *mind-independent* entity. Thus, whatever it is for which Chalmers (1996) and others now claim a subjective or phenomenal aspect, it cannot be what everyone else calls “information.”

A second objection to the Chalmers proposal, which this time he does acknowledge, is that thermostats (for example) clearly carry information, but are not widely regarded as having any degree of consciousness. Chalmers offers a choice of two options to deal with this:

1. Perhaps only some *kinds* of “physically realized information spaces” are conscious.
2. Perhaps thermostats are conscious.

Chalmers himself chooses option (2). He suggests, on no particular grounds, that the level of organization at which consciousness “winks out” might be lower than a thermostat but higher than a rock.

## Tononi's process theory

Another widely cited process theorist is Giulio Tononi. Tononi prefers Chalmers' option (1)—his integrated information theory (IIT) proposes that only *integrated* information is conscious. Actually the initial formulation of IIT (Tononi, 2004) sidesteps the question altogether, saying only: “The theory presented here claims that consciousness *has to do with* the capacity to integrate information” and “To recapitulate, the theory claims that consciousness *corresponds to* the capacity to integrate information.” [emphases added]. But this unobjectionable formulation soon morphs into the firm statement “consciousness is integrated information” (Tononi, 2008, 2012). Integrated information is defined in terms of various brain processes known to be associated with consciousness—one might almost infer that it was tempting simply to equate integrated information with conscious information, except that this would not have been terribly informative in the cybernetic sense of the word—and both Tononi and Seth et al. (2011) invest considerable effort in suggesting how integrated information might be quantified. Later Koch (2014) adds Chalmers' option (2) to the IIT mix and invokes panpsychism, admitting that inasmuch as integrated information is everywhere, consciousness must also be everywhere. Despite all the work that has by now been put into mathematical quantification of integrated information, no specific estimate of the quantity necessary for the appearance of consciousness is offered, but Koch speculates that the internet might be conscious.

## Mcfadden's process theory

McFadden (2013) in his CEMI (conscious electromagnetic information) theory, sticks with Chalmers' option (1), proposing that consciousness is associated only with *electromagnetically encoded* information. McFadden draws a distinction between extrinsic information (which he says is symbolic and arbitrary and exemplified by Shannon information) and intrinsic information, (which “preserves structural aspects of the represented object and thereby maintains some gestalt properties of the represented object”). He argues that “to avoid the necessity of a decoding homunculus, conscious meaning must be encoded intrinsically—as gestalt information—in the brain.” The precise relationship of this encoded gestalt information to consciousness is never spelled out, but it is probably not identity. McFadden does ascribe properties to consciousness and as he rightly says in his discussion of Chalmers' dual aspect theory, “it is not at all clear whether it is legitimate to ascribe properties to abstractions, such as the informational content of matter.”

## What's the problem?

There are several problems with all of this. First, since information is explicitly defined by everyone *except* process theorists as an objective entity, it is not clear how process theorists can reasonably claim either that information in general, or that any subset or variety of information in particular, is subjective. No entity can logically be both mind-independent and the very essence of mind. Therefore, when process theorists use the word “information” they must be talking about something quite different from what everyone else means by that word. Exactly what they are talking about needs clarification.

Second, since information is specifically defined by everybody (including Chalmers) as an abstract entity, any particular physical realization of information does not count as information at all. A “physical realization of an information space” like James Joyce's copy of Dante's Inferno may *carry* information, but it is not itself information—it's just an arrangement of paper and ink. A “physical realization of an information space” like Joe Bloggs' brain when he looks at an octopus may *encode* information, but it is not itself information—it's just an arrangement of neurons, glia and ions. Of course, it is certainly possible to claim that particular arrangements of neurons, glia and ions *are* conscious—indeed some remarkably eminent people have done so. But that claim no longer represents a dualist/process theory. It now represents a physicalist/vehicle theory. Since at least Chalmers specifically identifies his theory as dualist, it is far from clear how he (or others) can claim even information status, never mind consciousness, for any particular kind of “physically realized information space.”

Third, it is a problem at least for scientists that process theories are untestable. The hypothesis that a particular brain process *correlates with* consciousness can certainly be tested empirically. But the only potentially testable prediction of theories that claim *identity* between consciousness and a particular kind of information or information processing is that this kind of information or information processing will be conscious no matter how it is physically instantiated. This is a feature of process theories that makes them very attractive to those who would like to build a conscious artifact out of hardware. The unspoken prediction is that all one has to do to create artificial consciousness is emulate the computations done by the brain in some manner—any physical instantiation will do. But suppose it were possible to build a piece of hardware that adequately reproduced the brain computations underlying a particular sensory experience. How could we know whether the result was conscious? Consciousness is such a private phenomenon that nobody can be 100% sure even that their human neighbors are conscious at any given moment. We know *we* are conscious. Other humans look and act more or less like us, so when they tell us they have a particular conscious experience, we give them the benefit of the doubt. But what about a bit of hardware? Even a novice software writer could produce a piece of code that typed “I feel hot” whenever a thermostat registered a high temperature, but not many people would believe the appearance of this message meant the thermostat was experiencing hotness. Hence, neither the idea that information or information processing is conscious, nor its logical extension panpsychism (the idea that everything is conscious), is in any obvious way testable.

Of course, that doesn't necessarily mean these ideas are untrue. It just means they are unscientific. It may be fine for philosophers to play with the idea that thermostats and computer networks are conscious, but scientists are usually constrained to dealing in testable hypotheses.

### Conflict of interest statement

The author declares that the research was conducted in the absence of any commercial or financial relationships that could be construed as a potential conflict of interest.
